# Adoption and diffusion of zoning bylaws banning fast food drive-through services across Canadian municipalities

**DOI:** 10.1186/s12889-018-5061-1

**Published:** 2018-01-15

**Authors:** Candace I. J. Nykiforuk, Elizabeth J. Campbell, Soultana Macridis, Daniel McKennitt, Kayla Atkey, Kim D. Raine

**Affiliations:** 1 School of Public Health, 3–300 Edmonton Clinic Health Academy, University of Alberta, 11405 87 Avenue, Edmonton, AB T6G 1C9 Canada; 2 Alberta Centre for Active Living, 2-131 University Hall, University of Alberta, 8840 114 St NW, Edmonton, AB T6G 2H9 Canada; 3 Alberta Policy Coalition for Chronic Disease Prevention, 4-343 Edmonton Clinic Health Academy, 11405 87 Avenue, Edmonton, AB T6G 1C9 Canada

**Keywords:** Policy diffusion, Policy adoption, Health promotion, Fast food, Drive-through services, Drive-thru, Zoning, Diffusion of innovations, Municipal bylaw

## Abstract

**Background:**

Healthy public policy is an important tool for creating environments that support human health and wellbeing. At the local level, municipal policies, such as zoning bylaws, provide an opportunity for governments to regulate building location and the type of services offered. Across North America, there has been a recent proliferation of municipal bylaws banning fast food drive-through services. Research on the utilization of this policy strategy, including bylaw adopters and adopter characteristics, is limited within the Canadian context. The aim of this study was to identify and characterize Canadian municipalities based on level of policy innovation and nature of their adopted bylaw banning fast food drive-through services.

**Methods:**

A multiple case history methodology was utilized to identify and analyse eligible municipal bylaws, and included development of a chronological timeline and map of adopter municipalities within Canada. Grey literature and policy databases were searched for potential adopters of municipal fast food drive-through service bylaws. Adopters were confirmed through evidence of current municipal bylaws. Geographic diffusion and diffusion of innovations theories provided a contextual framework for analysis of bylaw documents. Analysis included assignment of adopter-types, extent and purpose of bans, and policy learning activities of each adopter municipality.

**Results:**

From 2002 to 2016, 27 municipalities were identified as adopters: six innovators and twenty-one early adopters. Mapping revealed parallel geographic diffusion patterns in western and eastern Canada. Twenty-two municipalities adopted a partial ban and five adopted a full ban. Rationales for the drive-through bans included health promotion, environmental concerns from idling, community character and aesthetics, traffic concerns, and walkability. Policy learning, including research and consultation with other municipalities, was performed by nine early adopters.

**Conclusion:**

This study detailed the adoption of fast food drive-through bylaws across Canada. Understanding the adopter-type characteristics of municipalities and the nature of their bylaws can assist other jurisdictions in similar policy efforts. While the implications for research and practice are evolving and dynamic, fast food drive-through service bans may play a role in promoting healthier food environments. Further research is required to determine the viability of this strategy for health promotion and chronic disease prevention.

## Background

Healthy public policy is an important tool for creating environments that support human health and wellbeing [1, 2]. At the local level, municipal policies, such as zoning bylaws, provide an opportunity for governments to regulate how lands and buildings are used, where buildings and services are located, and for what purposes they serve [3, 4]. For example, municipal zoning bylaws can regulate construction, location, and number of fast food restaurants within a given area [2]. Consequently, municipal zoning bylaws also allow for stipulations regarding fast food restaurants types and services, including drive-through facilities, highlighting the potential of such municipal policies to foster healthier food environments [3–6]. This has implications for public health as fast food restaurants have been highly scrutinized for serving high fat and energy-dense foods, which are low in nutritional value and have been linked to overconsumption and weight gain [1, 2]. In addition, fast food drive-through services provide a convenient and easily accessible way for individuals to purchase and consume foods without exiting their vehicle, also contributing to physical inactivity and distracted driving [5, 7].

In recent years, municipalities across North America have implemented zoning bans on fast food restaurants and drive-through facilities. While a number of jurisdictions have done so with the goal of fostering healthier food environments, in many cases, the rationale for policy adoption extends beyond health. The most commonly proposed reasons for such policies included: promoting health [4, 5, 8–16]; maintaining visual appeal of the community [4, 5, 10–12, 17–19]; addressing noise, safety and traffic concerns [17–22]; protecting local economy [4, 5, 23]; environmental considerations [5, 18]; addressing noise concerns; and enhancing community walkability [5]. As an example, in 1981, the Town of Concord, Massachusetts, in the United States banned fast food restaurants and drive-in services as a means to reduce traffic congestion, as well as to preserve and enhance the natural quality of the community [3]. Similarly, in 2008, the City Council in South Los Angeles, California, unanimously passed a regulation prohibiting the establishment of new stand-alone fast food restaurants and drive-through services as a means to encourage healthier food options [4, 13].

This study is timely because of the recent proliferation of fast food drive through bans, particularly in North America. In the last several years, fast food related bylaws, restrictions, and/or policies have emerged as a topic of interest in Canada, specifically pertaining to the adoption of municipal bylaws banning fast food drive-through services. However, little is known regarding the extent and spread of these bylaws among Canadian municipalities. Further, to date, no systematic reviews have examined the impact of food-related zoning laws and health outcomes in Canadian jurisdictions [24]. Moving forward, it will be important for public health researchers and policy makers to study and evaluate fast food drive-through service bylaws across different contexts and jurisdictions. Specifically, the application of policy diffusion theory, such as Rogers’ diffusion of innovations theory [25], allows for cross-jurisdictional learning of the various processes of testing and adopting innovation strategies across municipalities in Canada.

There is a critical knowledge gap in the Canadian context about the processes underlying development and adoption of fast food service related zoning bylaws to address health outcomes. As a means to address this gap and to foster cross-jurisdictional learning, the aim of this study was to identify municipalities across Canada that have adopted a bylaw that bans fast food drive-through services, as well as to characterize these adopter municipalities by the level of policy innovation and the nature of their adopted bylaw.

## Methods

### Study design

We adopted a multiple case study methodology to identify and characterize: (i) adopter municipalities across Canada, and (ii) the nature of the adopted fast food drive-through service bylaws. Case studies are a method used to investigate real world phenomenon by asking ‘how’ and ‘why’ [26]. A multiple case study design “refers to case study research in which several instrumental bounded cases are selected to develop a more in-depth understanding of the phenomena than a single case can provide” (p.582) [26]. A multiple case study methodology was therefore an appropriate study design to engender an in-depth understanding of the phenomenon of fast food drive-through service bans within a real-life context of multiple municipalities.

In this study, a ‘case’, referred to hereafter as ‘adopter’, was defined as a Canadian municipality that had adopted a ban on fast food drive-through services within a municipal zoning bylaw. The bylaw had to be enacted and publicly available at the time of this study (up to February 2016). Adoption was determined based on evidence of a formal bylaw, policy, official community plan, council resolution, or amendment document outlining a partial or full ban on new construction or building restrictions specific to fast food drive-through services in the municipality.

### Theoretical approach

Diffusion of innovations theory [25] provided an organizing conceptual framework for bylaw analysis and characterization of adopters. This theory proposes: key characteristics of adopters (i.e., their level of innovativeness based on five types and as reflected by their position in a diffusion curve); attributes of the innovation; and key contextual factors, such as communication channels and social networks, which are strong determinants of successful adoption. It outlines five types of innovation adopters: innovators, early adopters, early majority, late majority, and laggards [25]. In a general sense, innovators are the first to try a new idea and adopt an innovation (e.g., a fast food drive-through ban); as such, they are known to be venturesome. Early adopters are described as respectable, in that later adopters often seek the opinion of the early adopter before adopting an innovation. Next are the early majority, who are viewed as deliberate. This group interacts with peers to learn about an innovation, but rarely lead adoption of an innovation. Late majority are skeptical of new ideas and often wait for their peers to pressure them into adoption. Finally, the traditional nature of laggards results in this group being the last to adopt an innovation, and some in this group may only adopt the innovation once required to do so.

According to the theory [25], when adopters are plotted by adoption date on a graph of time against percentage of adopters, the resultant curve forms an S-shape. The position of an adopter on the curve defines their adopter-type based on the normal adoption distribution: innovator (2.5% of all adopters); early adopters (13.5%); early majority (34%); late majority (34%); and laggards (16%). In some cases, it may not be possible to plot policy adoption date on an S-shaped curve due to a small sample size [27]. Instead, researchers may consider both the date of adoption and characteristics of adopter innovativeness (as evidenced by government reports and examples of similar innovative initiatives) when assigning one of the five adopter-types.

### Data collection

#### Identification of municipalities

Two reviewers carried out a systematic search for municipal bylaws, restrictions, and/or policies on fast food drive-through services across Canada. The search involved looking for relevant grey literature using the Google search engine, Restaurants Canada website, various Canadian municipal government websites, University of Alberta Library Canadian Newsstand Complete, and Quick Law (for municipal board orders and decisions) to identify possible municipal bylaws within each province and territory across Canada. An array of documents, such as municipal council meeting minutes and newspaper articles, were also identified by hand searching reference lists of identified sources. Where the aforementioned documents identified other municipal jurisdictions as referents, snowball sampling was employed to identify relevant documents from those newly identified municipalities. The initial search yielded documents from a total of 130 municipalities across 11 provinces and territories. The search was conducted in June 2015, and performed again in February 2016 for updates.

### Identification of adopters

Adopters were identified by reviewing the source documents (i.e., copies of policies and bylaws, municipal council meeting minutes, and official community plans) to determine if: i) a fast food drive-through service ban had been considered and discussed by municipal council; and, if yes, ii) a bylaw, restriction, and/or policy or amendment was adopted. A municipality was characterized as an ‘adopter’ and included in the study if it met both criteria.

To identify adopters, coders followed four key steps. First, coders examined a municipality’s zoning bylaw to identify: the definition of a drive-through; general provisions of the zoning bylaw; and if these zoning bylaws were adopted and/or amended. Second, in cases where there was no indication of a ban within the zoning bylaw, coders then identified drive-through related policies within a municipality’s official community plan. If a ban could not be identified in the first two steps, step three was employed. Step three required coders to refer to news articles that initially identified a municipality as a potential adopter. News articles provided a source of key dates surrounding council discussion on the topic. This, in turn, informed a search within public council meeting minutes. Minutes were read to follow the decision-making process until a ban was adopted or rejected, allowing for confirmation. Finally, in cases where coders were still uncertain of adoption, municipal zoning bylaw officers and planners were contacted for clarification.

### Data analysis

#### Characterization of adopters

As described above, diffusion of innovations theory [25] categorizes adopters into one of five adopter-types based on the relative time at which they adopt a new idea in a network or community of adopters. According to the theory, when adopters are plotted by adoption date on a graph of time against percentage of adopters, the resultant curve forms an S-shape. The position of an adopter on the curve defines their adopter-type based on the normal adoption distribution: innovator (2.5% of all adopters); early adopters (13.5%); early majority (34%); late majority (34%); and laggards (16%).

Due to the limited number of municipalities identified as adopters in this study (likely due to the relative recent emergence of this policy option), an adoption curve could not be empirically fitted on an S-shaped curve. Therefore, each identified adopter municipality was plotted chronologically on a timeline and on a scatter plot using IBM SPSS Statistics 23 to examine policy spread. The timeline and scatter plot was then examined for trends and natural clustering of dates. Clusters of dates were grouped into adopter-types, with the innovator group having the earliest dates of adoption, followed by early adopters, early majority, and so on.

To confirm the adopter-type assignment of each municipality, two coders independently categorized each municipality using the diffusion of innovations theory definitions and key characteristics for each of the five adopter-types. Data used for this confirmation included municipal council reports outlining initial dates of policy discussion and development, newspaper articles highlighting public concerns with drive-through services, and personal communication with municipal planners and city clerks to clarify information and dates in public documents. Following the independent adopter-type assignment, the two coders and a third researcher discussed the findings. There was 100% agreement on adopter-type assignments.

### Characterization of nature of bylaw

To analyze the nature of the adopter’s bylaw, four characteristics were considered: geographic location of adopters; extent of ban (full or partial); bylaw intention or justification; and adopter policy learning activities (e.g., public consultation and research).

First, the geographic location of each adopter municipality was plotted on a map of Canada to identify possible geographical patterns. Adopters were plotted by province/territory and by region. For the purposes of this analysis, three regions were considered: eastern Canada region (comprising the following provinces: Newfoundland & Labrador, Prince Edward Island, Nova Scotia, New Brunswick, Québec, and Ontario); western Canada (Manitoba, Saskatchewan, Alberta, and British Columbia); or northern Canada (Nunavut, Northwest Territories, and Yukon).

Second, each adopter’s bylaw was reviewed to categorize the extent of the bylaw as either a ‘full’ or ‘partial’ ban of fast food drive-through services across municipal zones. A bylaw was considered a ‘full’ ban if the zoning bylaw banned the future construction of fast food drive-through services across all municipal zones. In some cases, this involved ‘grandfathering’ in existing facilities, rather than requiring the closure of operating drive-through services. A ‘partial’ ban described zoning bylaws that banned fast food drive-through services in one or more (but less than all) zones within the municipality. For example, the bylaw may ban drive-through services in residential and downtown zones, but allow them in highway commercial zones.

Third, the intent of each adopter’s bylaw was identified and assessed for alignment with bylaw intentions identified in the literature. Bylaw intentions identified in the literature included, in order of prevalence, related to: obesity and chronic disease [4, 5, 8–16]; protection of community aesthetics and character [4, 5, 10–12, 17–19]; traffic concerns [17–22]; safety [5, 10, 19, 20, 22]; reducing physical inactivity and sedentary behaviour [5, 8, 14, 28]; protecting local economy [4, 5, 23]; improving community nutrition [5, 9, 13]; air pollution, idling, and environmental concerns [5, 18]; decreasing inequalities by decreasing the density of fast food drive-through in low-income neighbourhoods [5, 29]; noise concerns from intercoms [19, 30]; and, improving community walkability [5].

Last, we identified policy learning activities where possible. These were grouped according to the typical policy learning activities of either public consultation or research activities. To be considered in the study, policy learning activities must have been conducted prior to policy adoption. Actions taken after policy adoption, though important, would not have influenced policy adoption and thus were beyond the scope of this study. Policy learning through public consultation included evidence of municipalities hosting public hearings to assess general interest and opposition to a potential drive-through zoning bylaw. Evidence of these hearings was found within municipal council meeting minutes and reports. Research activities included the municipality undertaking: i) a local inventory scan of fast food drive-through services and reviewing current zoning definitions and regulations; and/or ii) external research to understand zoning definitions and regulations of other municipalities across Canada.

## Results

### Adopters

**Fig. 1:**
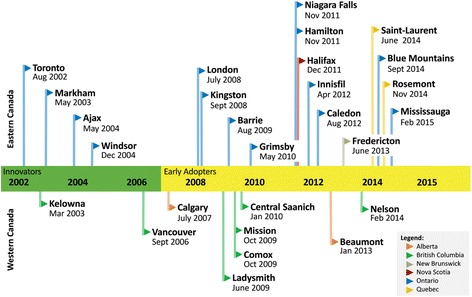
Municipal bylaw fast food drive-through bans by date of adoption, municipality, province, region, and adopter-type

Between January 2002 and February 2016, a total of 27 municipalities from six provinces were identified as adopters of fast food drive-through service bans through the adoption of a bylaw, bylaw amendment, or an official community plan (Table 1; Fig. 1). The identified set of adopter municipalities represents 0.7% of all Canadian municipalities (*N* = 3669) [31] and 24% of the Canadian population (*N* = 33,476,688) [32]. Of the 27 adopter municipalities, six (22.2%) were identified as innovators and 21 (77.8%) as early adopters (Table 1).

**Table 1:** Summary of Canadian municipal zoning bylaws banning fast food drive-through services

Region^a^	Municipality	Bylaw	Date of Adoption	Partial or Full Ban	Adopter-Type
Eastern	Toronto, ON	Zoning Bylaw No. 569–2013	26-Aug- 2002	Partial	Innovator
Eastern	Markham, ON	Zoning Bylaw Amendment Bylaw 2003–151	27-May- 2003	Partial	Innovator
Eastern	Ajax, ON	Bylaw Amendment No. 65–2004, to amend Zoning Bylaw No. 95–2003	10-May- 2004	Partial	Innovator
Eastern	Windsor, ON	Bylaw Amendment 375–2004, to amend Zoning Bylaw No. 8600	21-Dec- 2004	Partial	Innovator
Eastern	London, ON	Zoning Bylaw Z.-1–081795	22-Jul- 2008	Partial	Early Adopter
Eastern	Kingston, ON	Bylaw Amendment No. 2015–82, OPA Number 29; amending Restricted Area (Zoning) Bylaw No. 8499	2-Sep-2008	Partial	Early Adopter
Eastern	Barrie, ON	Zoning Bylaw-2009-141	10-Aug-2009	Partial	Early Adopter
Eastern	Grimsby, ON	Zoning Bylaw No. 14–4	3-May-2010	Partial	Early Adopter
Eastern	Niagara Falls, ON	By-law No. 2011–137	14-Nov-2011	Partial	Early Adopter
Eastern	Hamilton, ON	Zoning Bylaw Amendment 11–276	16-Nov-2011	Partial	Early Adopter
Eastern	Halifax, NS	Downtown Halifax Land-Use Bylaw	13-Dec-2011	Partial	Early Adopter
Eastern	Innisfil, ON	Zoning Bylaw Amendment - Bylaw 050–12	18-Apr-2012	Partial	Early Adopter
Eastern	Caledon, ON	Bylaw Amendment No. BL-2012-094, to amend Comprehensive Zoning Bylaw 87,250	14-Aug-2012	Partial	Early Adopter
Eastern	Fredericton, NB	Zoning Bylaw No. Z-5	24-June-2013	Partial	Early Adopter
Eastern	Saint-Laurent, QC	Le Reglement RCA08–08–0001-17	18-Jun-2014	Partial	Early Adopter
Eastern	The Blue Mountains, ON	Official Community Plan, 2014	3-Sep-2014	Partial	Early Adopter
Eastern	Rosemont-La-Petite-Patrie, QC	Amendment of the Planning Regulations Rosemont-Petite-Patrie (01–279); Rosemont-la-Petite-Patrie (01–279-39)	3-Nov-2014	Full	Early Adopter
Eastern	Mississauga, ON	Zoning Bylaw Amendment 0018–2015	11-Feb-2015	Partial	Early Adopter
Western	Kelowna, BC	Zoning Bylaw No. 8964 - Text Amendment No. TA02–0006	18-Mar-2003	Partial	Innovator
Western	Vancouver, BC	Zoning & Development Bylaw No. 3575, 2006 amendments	12-Sep-2006	Partial	Innovator
Western	Calgary, AB	Land Use Bylaw 1P2007	23-July-2007	Partial	Early Adopter
Western	Ladysmith, BC	Bylaw No. 1691	1-Jun- 2009	Full	Early Adopter
Western	Comox, BC	Rezoning Application RZ 09–2; Bylaw No. 1636	21-Oct-2009	Partial	Early Adopter
Western	Mission, BC	Zoning Bylaw 5050–2009	30-Oct-2009	Full	Early Adopter
Western	Central Saanich, BC	Land Use Bylaw Amendment 1667	11-Jan-2010	Full	Early Adopter
Western	Beaumont, AB	Land Use Bylaw 796–12	23-Jan-2013	Partial	Early Adopter
Western	Nelson, BC	Zoning Bylaw No. 3199, 2013 (2014/019)	2-Feb-2014	Full	Early Adopter

^a^Eastern region includes the following provinces: Newfoundland & Labrador (NL), Prince Edward Island (PEI), Nova Scotia (NS), New Brunswick (NB), Québec (QC), and Ontario (ON). Western region includes: Manitoba (MB), Saskatchewan (SK), Alberta (AB), and British Columbia (BC). Not all provinces in each region had a ban at the time of this study. There was also no evidence of municipal drive-through bylaws in the northern territories (Yukon, Northwest Territories, and Nunavut)

### Nature of bylaws

#### Geographic location of adopters

**Fig. 2:**
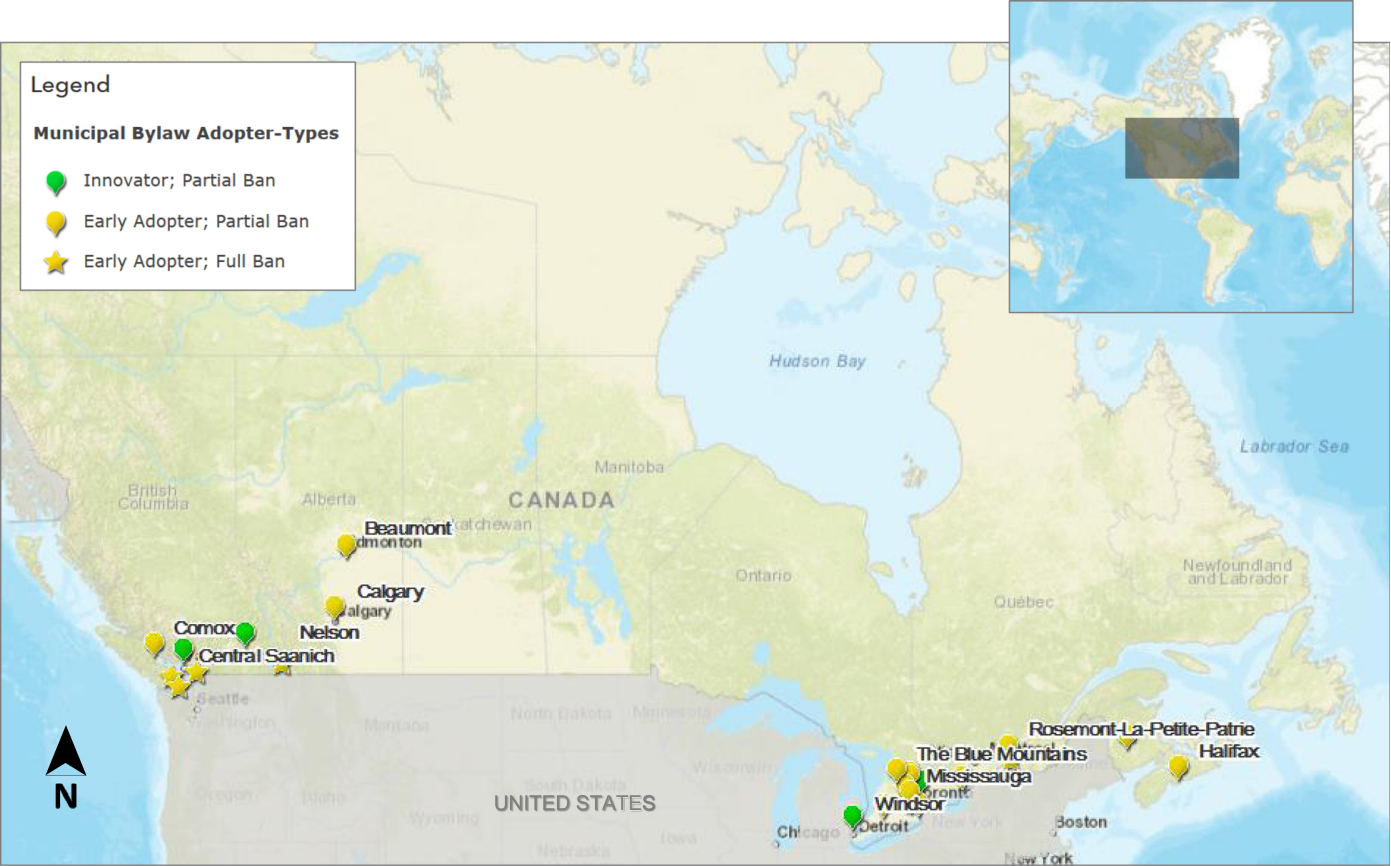
Map of municipal bylaw adopters across Canada by adopter-type. Map source: Esri, HERE, Garmin, NGA, USGS | Sources: Esri, HERE, DeLorme, Intermap, increment P Corp., GEBCO, USGS, FAO, NPS, NRCAN, GeoBase, IGN, Kadaster NL, Ordnance Survey, Esri Japan, METI, Esri China (Hong Kong), swisstopo, MapmyIndia, © OpenStreetMap contributors, and the GIS User Community

After plotting adopters on a map of Canada, we discovered two parallel geographic patterns of diffusion, one in eastern Canada and one in western Canada (Fig. 2). Of the total 27 adopter municipalities, 18 (66.7%) were located in the eastern region of Canada and nine (33.3%) in the western region of Canada (Table 1; Fig. 1). There were no adopters in the northern region of Canada at the time of the study. Accordingly, study findings are presented by region. The following sub-sections describe the chronological adoption of bylaws banning fast food drive-through services in municipalities, first in the eastern region of Canada, followed by adopters in the western region.

#### Eastern Canada

The first adoption of a fast food drive-through ban in Canada occurred in eastern Canada. In August 2002, Toronto, ON was the first municipality to formally adopt a bylaw to ban fast food drive-through services within the city [33]. In subsequent years, municipalities neighbouring Toronto, such as Markham, ON [34, 35], Ajax, ON [36], and Windsor, ON [37] adopted similar bans. Beginning in 2008, the cities of London and Kingston in Ontario both adopted fast food drive-through bans [38, 39]. This was quickly followed by the Ontario towns of Barrie [40] and Grimsby [31] in 2009 and 2010, respectively. Fast food drive-through bans continued over the next 5 years, with Niagara Falls, ON [41]; Hamilton, ON [42]; Halifax, NS [43] adopting bylaws in 2011. In 2012, Innisfil, ON [44, 45] and Caledon, ON [46] both adopted fast food drive-through bans. In 2013, another Atlantic city adopted a bylaw, this time in the City of Fredericton, NB [47]. Lastly, a proliferation of bylaw adoptions to ban fast food drive-through services was observed between 2014 and 2015. These included the borough of Saint-Laurent, QC [48, 49]; The Blue Mountains, ON [50, 51]; the borough of Rosemont-La-Petite-Patrie, QC [52]; and Mississauga, ON [53].

#### Western Canada

In western Canada, adoption of bylaws banning fast food drive-through services began with Kelowna, BC in 2003 [38] and the City of Vancouver, BC [39, 54] in 2006. Both municipalities amended their zoning and development bylaws in order to ban and restrict fast food drive-through services in their municipalities. Adoption of fast food drive-through bans continued in western Canada spanning the provinces of British Columbia and Alberta. The first Albertan municipality to adopt a fast food drive-through ban was Calgary, AB [55] in 2007. Subsequently, the municipalities of Ladysmith [56], Comox [57], and Mission [58] in British Columbia each adopted bylaws in 2009, followed by the District of Central Saanich, BC in 2010 [59]. Most recently, in 2014 Beaumont, AB [60] and Nelson, BC [77, 78] succeeded in adopting fast food drive-through bans.

#### Extent of bans

Fast food drive-through service bans were categorized either as a ‘full’ or ‘partial’ ban based on the number of zones affected. A full ban categorized municipalities that banned fast food drive-through services across the entire municipality, while partial bans applied to one or more (but less than all) municipal zones. Our categorization of municipal bylaws revealed that 22 (81.5%) municipalities adopted a partial ban and five (18.5%) municipalities adopted a full ban (Table 1).

#### Intent of bans

We compared the purposes and rationalizations for fast food drive-through service bans and policies from our study to the intentions of similar policies as identified in the literature (Table 2). Study findings pertaining to bylaw intent aligned with eight of the 11 themes identified in the literature, with many bylaws covering multiple intentions. The intentions identified in our study that aligned with the literature include the following: protect community aesthetics and character; reduce traffic concerns and reduce dependency on automobiles; protect community comfort and safety; reduce physical inactivity and sedentary behaviour; protect local economy; address environmental concerns by reducing air pollution and idling; address noise concerns from intercoms; and promote community walkability, active transportation, and public transportation. Our study revealed four additional fast food drive-through bylaw intentions not found in the literature: improve urban design and promote downtown centres; promote sustainability and sustainable development; reduce odour and litter in neighbouring communities; and reduce visual impact of drive-through facilities, including lighting or sound encroachment in neighbouring communities. Of note, no municipalities identified in our study outlined ‘obesity and chronic disease’ as a reason to adopt a fast food drive-through service ban, although the literature identified this intent as the most common reason for a ban on fast food drive-through facilities.

**Table 2:** Summary of intentions and rationales identified in the literature for adopting a zoning bylaw banning fast food drive-through services, compared to bylaw intentions identified in this study

Bylaw intentions identified in literature	Bylaw intentions identified in this study
Obesity and chronic disease [4, 5, 8–16]	Not identified
Protect community aesthetics and character [4, 5, 10–12, 17–19]	Protect community aesthetics and character (*n* = 10)
Traffic concerns [17–22]	Traffic concerns and reduce dependency on automobiles (*n* = 10)
Safety [5, 10, 19, 20, 22]	Community comfort and safety (*n* = 4)
Reduce physical inactivity and sedentary behaviour [5, 8, 14, 28]	Reduce physical inactivity and sedentary behaviour (*n* = 1)
Protect local economy [4, 5, 23]	Protect local economy (*n* = 1)
Improving community nutrition [5, 9, 13]	Not identified
Air pollution, idling, and environmental concerns [5, 18]	Air pollution, idling, and environmental concerns (*n* = 9)
Reduce inequalities by decreasing the density of fast food drive-through in low-income neighborhoods [5, 29]	Not identified
Noise concerns from intercoms [19, 30]	Noise concerns from intercoms, especially near residential areas (*n* = 7)
Improve community walkability [5]	Promote community walkability, active transportation, and public transportation (*n* = 15)
Not identified	Urban design, promote downtown core (*n* = 10)
Not identified	Sustainability and sustainable development (*n* = 2)
Not identified	Reduce odour and litter (*n* = 1)
Not identified	Reduce visual impact of drive-through and lighting/illumination encroachment, especially near residential areas (*n* = 4)

#### Policy learning

Of the 27 adopters, nine municipalities (33.3%) undertook one or more policy learning strategies to inform the process of adopting a bylaw banning fast food drive-through services. Policy learning was categorized as public consultation and/or research (local scan or on external policies). All nine municipalities that undertook one or more policy learning activities were characterized as early adopters, and none of the six innovator municipalities demonstrated evidence of performing policy learning activities. Of the nine early adopters that took action, six municipalities (66.7%) conducted both public consultation and research activities (local scan: *n* = 5; external research: *n* = 1). Of the remaining three, one municipality (11.1%) conducted only public consultation, and two (22.2%) conducted only research (local scan: *n* = 1; external research: *n* = 1) as part of their policy learning activities. There was no documented evidence of policy learning strategies utilized by the remaining 12 early adopter municipalities.

## Discussion

This is the first study to examine municipalities across Canada that have adopted a zoning bylaw that bans fast food drive-through services. Over a 13-year period, very few municipalities across Canada have adopted a ban on fast food drive-through services. Of the adopters, we observed two parallel geographic patterns of fast food drive-through service ban adoptions: one in eastern Canada and one in western Canada. Densely populated municipalities within eastern Canada, namely the city of Toronto, ON, led the way with diffusion spreading the policy to surrounding municipalities. This finding aligns with the notion that innovators tend to be larger, more populous, and wealthier [61] than the typically lesser-populated jurisdictions in the early adopter and later adopter groups.

In western Canada, the pattern of diffusion was similar, with bylaw spread likely initiating with a partial ban adopted by Kelowna, BC (2003), and followed by partial bans in the densely-populated cities of Vancouver, BC (2006) and Calgary, AB (2007). However, it was the smaller municipalities in western Canada that were the first to adopt full fast food drive-through bans (Table 1). These findings are consistent with a previous study of US municipal fast food restaurant land-use regulations [4], which found that most adopter municipalities were of relatively small population size. Of the 77 municipalities included in the study, approximately 56% had a population of less than 20,000 people (and approximately 40% with a population of less than 10,000) [4]. These findings suggest that there may be benefits and facilitators beyond large population size and wealth that influenced the adoption of fast food drive-through services in some Canadian municipalities.

Awareness of adopter characteristics is critical to a complete understanding of how policies diffuse, particularly if one is seeking to leverage or enhance the policy cycle to achieve a particular policy outcome. Herein, we identified two of Rogers’ five adopter-types [25]: innovators and early adopters. The remaining three adopter-types were not present at the time of study, likely due to the large number of potential adopters that have yet to adopt a fast food drive-through bylaw. Adopters of novel or emerging policies often do not include all five of Rogers’ diffusion of innovations adopter-type categories, as adoption by all groups may take considerable time to fully play out [62].

According to Rogers’ Diffusion of Innovations Theory [25], innovators tend to be more cosmopolite (i.e., larger urban or metropolitan areas), are more willing and often eager to try new ideas, have a greater threshold for risk, have a larger population with a wealth of resources, have extensive social networks, and are first to adopt a new innovation. In our study, innovators in eastern Canada were large cosmopolitan municipalities with many resources available to take risks with new innovations. However, in western Canada, there was a mix of population sizes among the innovators. This finding of varied diffusion patterns across Canada aligns with research on smoke-free policies in the provinces of Ontario and Alberta [62]. In both provinces, diffusion occurred both down hierarchies from larger, urban cities to less populated municipalities, and upwards from smaller centres to larger municipalities, with innovators representing a variety of population sizes.

Beyond innovators, Rogers characterizes early adopters as being more integrated within a local social system than innovators, making them localities [25]. To remain respected, early adopters must ensure that a judicious decision-making process is undertaken before adopting a new idea. In essence, the early adopter’s role is to reduce the amount of uncertainty for other jurisdictions to facilitate adoption of an innovation [25]. Adoption of a new idea often involves a level of learning and understanding of whether a policy adopted elsewhere was successful [61]. This characterization aligns with the characteristics of the early adopter municipalities identified in our study, particularly in terms of the policy learning activities that were taken by this group. For example, over 40% of early adopters in the current study conducted research and/or public consultation activities, compared to none of the innovators. The early adopters were more informed during their decision-making process and considered some evidence prior to adoption. In addition, the early adopters did not copy previous bans, but rather used their policy learning strategies to implement a ban to fit the needs of their municipality.

Due to the emergent nature of this policy topic, we have yet to see how the early adopters may influence later adopter-types to move forward with a fast food drive-through ban. Research indicates that diffusion may take a number of decades [62], and furthermore, may be delayed by what is known as a ‘chasm’ between the early adopter and early majority groups [63, 64]. The chasm describes the rapid adoption of innovations (in this case, fast food service drive-through bans), followed by a ‘lull’ as the innovation either is discontinued or evidence of its efficacy encourages adoption by the early majority adopter group [64]. The ‘chasm’ presents a timely opportunity for healthy public policy advocates to advance future policy adoption by assessing policy impact of extant policies and actively disseminating findings of policy efficacy to potential early majority adopter municipalities.

The focus of our study was to identify and characterize current adopters of municipal fast food drive-through service bylaws in Canada. Beyond the 27 municipal adopters, we also identified two groups of non-adopters: those who attempted to adopt a ban and failed; or those who were currently in the process of bylaw adoption. Similarly, a study of US bylaws regulating fast food restaurants and drive-through services in the United States found that of the 100 included policies, 63 were enacted, five were enacted and then later repealed, and 32 were raised in council but were not passed [4]. It is therefore important to understand the reasons why some municipalities were unsuccessful with their policy efforts, as well as understand why others may take longer to adopt.

Examples of Canadian municipalities that attempted to adopt a bylaw, but failed include Squamish, BC and Côte-des-Neiges—Notre-Dame-de-Grâce, QC. In both cases, the municipality was undertaking the bylaw development process around the same time as nearby successful adopters. However, due to involvement from opposition groups, such as the Restaurants Canada (formerly known as Canadian Restaurants & Foodservices Associations (CRFA)), both municipalities failed to adopt a bylaw [65, 66]. Although this study may not capture all reasons for failure to adopt, other identified reasons found in the study data included competing policies or alternate directions (e.g., anti-idling bylaws) and/or lack of political support. Further investigation of failed attempts to adopt fast food drive-through services bans may help those interested in such a policy to address the identified barriers, better engage with key stakeholders, and inform decision-makers of the policy change efforts already put forward. Together, these actions may ignite commitment to persist in successful adoption.

At time of this study, there was evidence that four municipalities were in the process of adopting a ban. For example, Waterloo, ON has included regulations on fast food drive-through service development as part of their Official Plan [67], with zoning bylaw amendments yet to be adopted, but anticipated. Similarly, in February 2016 the municipal council of the Corporation of Delta, BC adopted a recommendation from the Community Planning Advisory Committee that the municipality develop guidelines and/or a policy relating to drive-through facilities [68]. These examples indicate that drive-through service bylaws and policies continue to be of focus in some municipalities across Canada. Furthermore, these potential adopter municipalities may represent early majority adopter-types, as per diffusion of innovations theory. These groups require more time to research their options, get buy-in from municipal decision-makers, and likely require evidence of successful adoption in other, similar jurisdictions before choosing to adopt a policy. It will be important for public health researchers and policy makers to track the progress of these majority group adopters as diffusion continues.

Also important are those municipalities that have adopted design guidelines or regulations to determine where drive-through services may be located and what design requirements they must meet. The City of Ottawa, Ontario, for example, developed urban design guidelines for drive-through facilities in 2006 [69]. Similarly, the City of St. John’s, NFLD developed drive-through facility regulations in 2012 that required all applications for a drive-through to be reviewed and approved by the city before development [70]. Unfortunately, many of the guidelines and plans do not have legal grounding, like that of a bylaw, and thus may not be fully enforced. These examples of guidelines and regulations are evidence that municipalities are taking action towards addressing an issue, but also demonstrates that municipalities such as these are not able or willing to adopt a more permanent, enforceable policy. These municipalities may only adopt a formal ban once the benefits of the policy clearly outweigh the risks, or after a large proportion on municipalities across Canada adopt fast food drive-through service bylaws. Further, considerations of issue framing are important: while health promotion and chronic disease prevention are public health gains from the implementation of fast food drive-through service bylaws, none of the successful adopters outlined this as part of their rationale for their bylaws. These cases have demonstrated that it may not be necessary to invoke a health frame in order to realize municipal healthy public policy.

### Strengths and limitations

To our knowledge, this study is the first to explore the adoption and diffusion of bans on fast food drive-through services in municipalities across Canada. While zoning and land-use bylaws banning fast food restaurants and drive-through services have been studied in the United States [4, 5, 12, 13, 15], there has been limited research conducted in the Canadian context. Our contribution to the literature may be useful to public health departments and local governments seeking to employ zoning bylaws, restrictions, and/or policies as a strategy to foster healthy food environments.

Application of the diffusion of innovations theory [25] was a useful framework in describing the spread of adoption of bylaws through adopter categories. This is novel as, to date, the diffusion of innovations theory framework has not previously been applied to the literature on zoning bylaws to address health outcomes. This may inspire future work in this area as an increased understanding of how policies spread is crucial to study healthy public policy adoption.

This study was limited to by the relative short period of policy activity (i.e., since 2002): currently, only innovator and early adopter categories were captured. As this policy issue continues to emerge, public health actors and other stakeholders can utilize the growing momentum to employ scholarly methods, such as longitudinal and case control studies, in an effort to better understand the adoption, spread, and impact of bylaws banning fast food drive-through services over time and across jurisdictions. Further, assessment of policy effectiveness, while beyond the scope of this study, is required to determine the viability of fast food drive-through bans strategy for health promotion and chronic disease prevention.

The short period of policy activity and limited number of adopters to date were also contributing factors to limitations in study design. For instance, the methods employed were constrained by the number of adopter municipalities. Our determination of adopter-types was based on the clustering of adoption dates and alignment with broad definitions for each adopter-type, rather than an empirical fit to Rogers’ diffusion S-curve [25], which was not possible with such a small number of adopters. Consequently, policy diffusion analysis and justification for adopter-type categorization becomes challenging when subjective constraints are present. However, this novel approach to characterizing adopter-types may be of merit for future research to develop a qualitative protocol for adopter-type characterization based on Rogers’ diffusion of innovations theory. Another possible solution to this limitation would be to re-examine this policy at a later point in time once more municipalities have adopted a fast food services drive through bylaw, or when related policy options emerge.

As policy adoption is on-going in nature, our list of adopters is inclusive only up to when data analysis occurred, and characterization of those adopters relative to others in the community or network of adopters may be subject to change over time. If examined again in another decade or two, and assuming continued adoption of this policy leading to a greater number of adopters, it is possible that those municipalities categorized as early adopters at this point in time may actually be seen as innovators relative to the rest of the adopters in the community. Further, investigation of the factors that influenced the diffusion of fast food services drive-through bylaws (e.g., policy opportunity windows, geographical location, and political climate) were beyond the scope of this study. However, such diffusion and contributing factors could be the foci of future studies.

## Conclusions

This is the first study to examine municipalities across Canada that have adopted a zoning bylaw that bans fast food drive-through services. This policy diffusion study detailed the chronological history, geographical patterns, and adopter characteristics of Canadian municipalities with fast food drive-through bans. The characterization of the adopter municipalities, along with the nature of the bylaw adopted, contributes a fuller understanding of the factors associated with bylaw adoption. In addition, while the policy cycle can be a complex and arduous process, this study demonstrated the utility of policy learning, as a characteristic of policy diffusion, to support the adoption of fast food drive-through bans. Municipalities motivated to adopt a fast food drive-through bylaw ban enhanced their policy development cycle by utilizing previously developed policy drive-through bylaw bans in similar jurisdictions, thereby saving time and resources.

Our study findings revealed a limited number of adopter municipalities, suggesting that policy diffusion of zoning bylaws for drive-through bans is still an emerging process. Further, as policy diffusion is in its infancy with regards to bans utilizing zoning bylaws, evaluations of current policies are required to examine the impact and effectiveness of bylaws banning drive-through services on population health and other indicators of interest to communities (e.g., economic or environmental outcomes).

Overall, the implications for research and practice in zoning bylaws for drive-through services are evolving and dynamic. Fast food drive-through service bans are one policy option that may be considered as part of a comprehensive, multi-pronged strategy to promote healthier food environments and improve population health. Research in the area of healthy food environments and zoning bylaw utilization may prove to be a vital part of preventing chronic disease in Canada.

## Abbreviations

AB: Alberta
BC: British Columbia
NB: New Brunswick
NS: Nova Scotia
ON: Ontario
QC: Québec
US: United States of America
